# A dawn and dusk chorus will emerge if males sing in the absence of their mate

**DOI:** 10.1098/rspb.2023.2266

**Published:** 2023-11-22

**Authors:** Lotte Schlicht, Emmi Schlicht, Peter Santema, Bart Kempenaers

**Affiliations:** ^1^ Max Planck Institute for Biological Intelligence, Eberhard Gwinner Str, 82319 Seewiesen, Germany; ^2^ Edward Grey Institute, Department of Biology, University of Oxford, Oxford, UK

**Keywords:** blue tit, *Cyanistes caeruleus*, dawn chorus, female behaviour, mate coordination, song rate

## Abstract

The spring dawn and dusk chorus of birds is a widespread phenomenon, yet its origin remains puzzling. We propose that a dawn and dusk chorus will inevitably arise if two criteria are met: (1) females leave their roost later in the morning and go to roost earlier in the evening than their mate, and (2) males sing more when separated from their mate. Previous studies on blue tits (*Cyanistes caeruleus*) support the first criterion. We here report that males sing at a higher rate whenever they are separated from their mate and that song rate increases with the duration of female absence. These findings can explain the existence of the dawn and dusk chorus in blue tits, and they can explain why the dawn chorus is more pronounced than the dusk chorus, as is typically observed. An exhaustive literature search provides support for both criteria of the ‘absent mate’ hypothesis in several passerine birds. We found no evidence contradicting the hypothesis. The new hypothesis is not inconsistent with many of the existing hypotheses about dawn singing, but may be a more general explanation for the occurrence of a dawn and dusk chorus. We describe how the ‘absent mate’ hypothesis leads to testable predictions about daily and seasonal variation in song output.

*It is not certain whether such singing should be regarded as due primarily to 'absence of the female' or to internal factors.* R. A. Hinde 1952, writing about the dawn chorus

## 1. Introduction

Across the globe, individuals of many bird species engage in a daily bout of synchronized singing in the early morning [1,2] and to a lesser extent also in the evening [2,3]. The resulting dawn and dusk choruses are among the most conspicuous and recognizable acoustic displays in the animal world [1,2] and an iconic symbol of nature and its conservation [4–6]. Theoretical and empirical studies have proposed and tested various non-mutually exclusive hypotheses to explain why males sing disproportionally at dawn [2,3]. Although there is some evidence for each of these hypotheses, none of them provides a universal explanation for this widespread phenomenon [2,3].

Here, we propose a new hypothesis for the existence of a dawn and dusk chorus based on a simple, universally applicable mechanism. The hypothesis posits that a singing peak in the early morning and in the late evening is the inevitable outcome if two criteria are met:

(1) most females leave their roost later in the morning and go to roost earlier in the evening than their social mate, and
(2) males are more likely to sing or sing at a higher rate when their social mate is absent.

**Figure 1.**
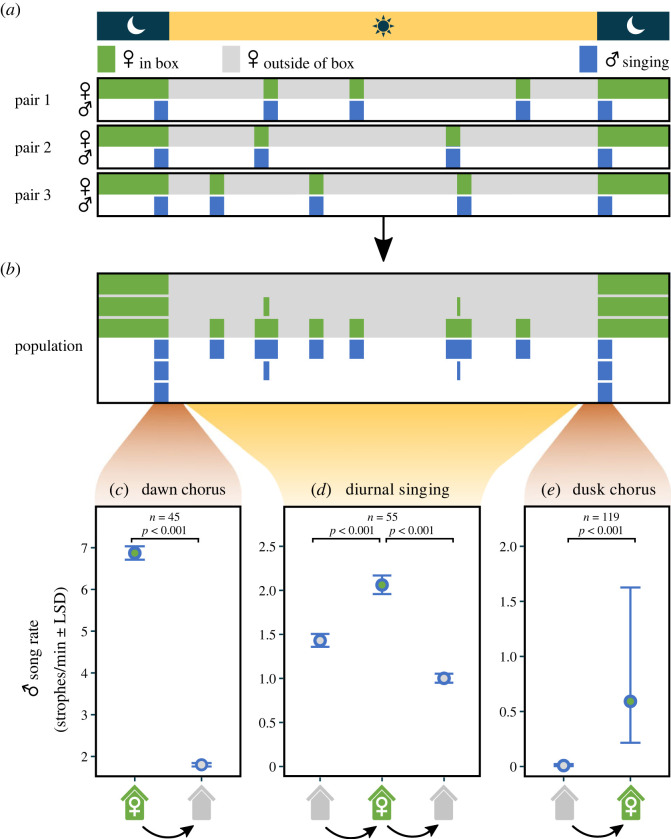
Illustration of the idea behind the absent mate hypothesis (*a*–*b*) and the corresponding results from this study (*c*–*e*). (*a*) Schematic illustration of three hypothetical breeding pairs. With the exception of their own roosting period (at night), males sing (blue) whenever their mate is at the nest or roost (here indicated in green) and stop singing when their female is away from the nest (grey). (*b*) At the population level, the behaviour of the three hypothetical breeding pairs from (*a*) will produce a dawn and dusk chorus, while song will be more sporadic during the day. (*c*–*e*) Blue tit males sing more when the female is inside the nest-box at dawn (*c*), during the day (*d*) and at dusk (*e*). Shown are estimates and least significant differences (LSD; see Methods), sample sizes (*n*) and *p*-values comparing male song rates (strophes min^–1^) 30 min before and after the female leaves the box at dawn (*c*), before, during and after the female is inside the nest-box during the day (*d*) and 10 min before and after the female enters the box to roost at dusk (*e*). Note the different scales of the *y*-axes. Poisson estimates of song rate are back-transformed to the original scale (strophes/minute). An example of the male and female behaviour of a single pair is shown in electronic supplementary material, figure S6.

If most males start their morning activity before their mate has left the roost and if this separation from the social partner induces them to sing, a dawn chorus will arise (figure 1*a,b*). The dawn chorus will end when all females have left the roost and joined their mate. Similarly, males will start to sing in the evening once their mate has gone to roost, resulting in a dusk chorus, which will end when the males go to roost themselves.

We test the ‘absent mate’ hypothesis by examining the validity of the two underlying criteria in a nest-box-breeding population of a common and well-studied passerine bird, the blue tit *Cyanistes caeruleus* [7,8]. Blue tits exhibit a pronounced dawn chorus and a less pronounced dusk chorus [9–13]. In the early breeding season males on average wake up and start their activity earlier in the morning than females, and females go to roost earlier than males in the evening [14,15], implying that criterion 1 is met in blue tits. To evaluate whether male blue tits sing more when their female is absent (criterion 2), we defined the ‘absence’ of the female as the time she spent alone inside a nest-box, and compared male song rate during this period with periods of equal length before the female entered the nest-box and after she left the nest-box. We examined the effect of female absence separately during dawn and dusk, and during the day, and we test whether male song rate increased with the duration of his partner's absence. Finally, we examined the literature to assess whether the two criteria that form the foundation of the ‘absent mate’ hypothesis may be generally valid in birds.

## 2. Methods

### (a) Study site

We studied a population of blue tits in the spring of 2019 in a mixed-deciduous, oak-dominated forest near Landsberg am Lech in southern Germany (‘Westerholz’; 48° 08′ 26″ N, 10° 53′ 29″ E). In the 40 ha study area, 277 nest-boxes have been monitored since 2007 as part of a long-term study on the breeding biology of blue tits. Each year 62 to 201 nest-boxes were occupied. Most females started their clutch in April (median first egg date: 17 April, range: 29 March–17 June) and females laid a median clutch size of 10 eggs (range 4–16). In blue tits, the female builds the nest and incubates alone, but the male may provide her with food [16,17]. Both parents provision the offspring [16]. To monitor the progress and content of nests, we checked nest-boxes at least weekly, and daily when the nest was finished and again close to hatching and fledging to determine the exact laying, hatching and fledging dates. We never observed a second breeding attempt after successful fledging of a brood, but replacement clutches occurred after the first attempt failed at an early stage. Polygyny occurred in the study area at a low rate (proportion of polygynous males; mean: 3%, range: 0–9%, [18]). Here, we excluded data from polygynous males and from known replacement clutches. More details of the study site and general field procedures are reported elsewhere [19].

### (b) General approach

We investigated the relationship between male singing and female absence for 76 socially monogamous breeding pairs. We determined across the entire day (1) when the female was alone inside a nest-box and (2) when the male was singing. Dawn song in blue tits is most pronounced close to the start of and during early egg laying [20]. We therefore used data from 5 days before to 5 days after the female laid her first egg. During this period, the male rarely enters the nest-box [16], and 70–80% of the females roost in the nest-box in which they lay their eggs [19]. Pairs do not roost together [14,16]. We therefore defined the female of a social pair as ‘absent’ (i.e. not with the male) when she was inside the nest-box, while the male was not [21,22]. This definition of female ‘absence’ is imprecise, because (1) the male may or may not be aware that his partner is inside the nest-box, and (2) pair members may also be separated when the female is not in the nest-box. This will lead to noise in the data, which may potentially conceal a relationship between male singing rate and female absence as defined here, but it will unlikely result in a false positive relationship.

### (c) Data on female presence in the nest-box

We used RFID technology to determine when the female of a pair was inside a nest-box and thus considered separated from the male. Each nest-box in the study site was equipped with a radio-frequency identification (RFID) reader and one light barrier at the outside and one at the inside of the entrance hole, such that the date and time and the direction of the movement of any individual with a passive integrated transponder (PIT) that entered or left the nest-box was recorded [23]. All females and males of the studied breeding pairs were equipped with a PIT, which was inserted under the skin on the back upon capture [24]. Birds had been caught during a previous breeding season inside a nest-box when they provisioned their offspring, or during a preceding winter with a mistnet.

Not all entries and exits could be unequivocally assigned, for example because the transponder failed to be recorded when a bird flew fast into the box. We therefore conservatively only included nest-box visits if we could reliably determine the entry and exit times using the information provided by the two light barriers during the entry and the exit. We verified the accuracy using video recordings for a subset of the nest visit data. We further only included female nest-box visits that lasted at least 2.5 min, because we only expect a behavioural reaction if the male notices the female's absence and because of practical reasons (see below). For a description of female nest-box visits see figure 1 in electronic supplementary material.

### (d) Data on male singing activity

We recorded the singing behaviour of males by placing sound recorders (Song Scope 4.1.3, Wildlife Acoustics, Concord, MA) at the base of each tree that carried a nest-box with an active nest. Each recorder had one microphone placed about 25 cm from the ground and pointing upwards. Male blue tits usually sing inside their territory or at the territory boundary (in 2019 the distance between two neighbouring occupied nest-boxes was 40–80 m) and an intruding male that sings is typically chased immediately [9] (see also electronic supplementary material, text S1). With each song recorder we therefore targeted the focal male of a pair, assuming that this male would produce most of the (louder) songs recorded at this location (see below). We programmed the sound recorders so that they recorded continuously each day from 2 h before sunrise to 30 min after sunset. During the study period (days −5 to +5 relative to the start of egg laying, day 0), males left their roosting site on average more than 20 min earlier in the morning and entered it about 10 min later in the evening than females (data re-analysed from [15]). From the recordings, we extracted all song (sonograms shown in electronic supplementary material, figure S4) during the following periods: (1) one hour around sunrise (30 min before and 30 min after), (2) 20 min around sunset (10 min before and 10 min after), (3) during the day, whenever the focal female was inside the nest-box (for at least 2.5 min), and during the corresponding periods of the same duration (as her stay inside) before she entered and after she left the nest-box (see figure 1*c* for an example). Details on the sound extraction and definitions are given in electronic supplementary material, text S2. In brief, one of us (LS) counted all blue tit vocalizations in each of the relevant periods that are considered ‘song’ (electronic supplementary material, figure S4). Song scoring was done blindly with respect to male and female identity, time of day, the day relative to laying start and whether or not the female was inside the nest-box at the time. For all scored vocalizations we also measured their loudness in dB from the spectrogram.

We used a loudness (dB) threshold to define whether a song was produced by the male of the focal pair. We assumed that vocalizations louder than the threshold were produced by the focal male and included them into the analysis, whereas quieter vocalizations could have been produced by a male on a neighbouring territory and were therefore excluded from the analysis. This approach leads to errors, because song from the focal male may be excluded (threshold too high) and song from non-focal males may be included (threshold too low). To assess and mitigate this problem, we used two approaches (details in electronic supplementary material, text S1). First, we observed the identity of singing males (focal or non-focal, based on colour bands) and estimated their distance to their nest-box, where a sound recorder was positioned. We then scored the loudness of the songs recorded by the sound recorder. Focal males sang on average at −31 dB (range: −15 to −39 dB), non-focal males sang on average at −44 dB (range: −26 to −53 dB), and the optimal loudness threshold should be in between those two averages (see electronic supplementary material, text S1). Second, for a subset of the recordings, we identified songs that overlapped in time and scored their loudness. Assuming that the louder of the two songs is from the focal male, we defined an optimal loudness threshold (−36 dB, see electronic supplementary material, text S1), on which all results in the main text are based. Third, we also used these data to define a range of loudness thresholds (from −40 to −30 dB), and we repeated all analyses using different thresholds within this range to test the sensitivity of the conclusions to variation in the loudness threshold. The results remained similar across the entire range of thresholds (electronic supplementary material, tables S1–S5), suggesting that the conclusions are robust and invariant to the exact choice of the threshold.

### (e) Statistical analysis

All statistical analyses were performed in R 4.1.3 [25]. All tests are generalized linear mixed effect models using a zero-inflated Poisson distribution with a log link function using the package glmmTMB [26]. We inspected the fit of the model assumptions using the package DHARMa [27]. For all models, we included the number of separate songs (strophes) during a given period as the response variable, and pair identity (nest-box) and the identity of the female nest-box visit event as random intercepts.

We first ran a series of models to test whether male song output is related to female absence. The explanatory variable is ‘period’, which is a three-level factor for recordings during the day (comparing periods of equal length before the female entered the box, while she was in the nest-box, and after she left the box), and a two-level factor for recordings around dawn (comparing song output 30 min before and 30 min after the female left the nest-box) and dusk (comparing song output 10 min before and 10 min after the female entered the nest-box). We used 30 min at dawn and 10 min at dusk, because these periods approximate the average time males are active before the female emerged at dawn and are still active after the female entered the roost at dusk (calculated from [15]). For all analyses of dawn, day and dusk data, female nest-box visits in which the ‘after’ period of one visit overlapped with the ‘before’ or ‘inside’ period of the next visit were excluded (excluded: *n* = 73 visits by 2 pairs; included: 119 visits by 74 pairs).

When considering differences in song output between periods (figure 1*c–e*), we show estimates and least-significant differences (LSDs) divided by two (following [28]). LSDs can be interpreted as ‘confidence intervals of a difference’ and show the minimum difference required for two (or more) estimates to be significantly different at *p* < 0.05. Thus, non-overlapping bars indicate a statistically significant difference between two (or more) estimates. For each given model, we calculated LSDs by multiplying the model estimate for the difference between song rates during different periods (before, during, after female nest-box visit) with the 97.5% percent quantile of a *t*-distribution (≈ 2) using the number of visits as the degrees of freedom.

In a second model we tested whether male song output increases with increasing duration of female absence during the day. In this model the explanatory variable is ‘time since female nest-box entry (in minutes)’ and the response variable is the number of songs per minute. In contrast to the previous models, here each datapoint refers to the song output during a one-minute period.

To test the robustness of the conclusions, we repeated all analyses using three additional model families (electronic supplementary material, tables S1–S5): Poisson (excluding data points without song, log-link function), binomial (response: song yes/no, logit-link function) and Gaussian (+0.01 and then log-transformed). All models yielded similar results, but the effects were stronger in the Poisson models than in the binomial models, suggesting that most of the variation is explained by the Poisson-part of the zero-inflated Poisson model presented in the Results. We also used different loudness thresholds to define song by the focal male, ranging from −30 to −40 dB, which did not lead to substantially different results (electronic supplementary material, tables S1–S5).

To ensure that the change in song rate during the day is not associated with behaviour specific to the female entering or exiting the nest-box (i.e. unrelated to the female's absence), we repeated the analyses after removing all data 2 min before or after female entry or exit.

### (f) Literature search

To evaluate the evidence in support of or contradicting criteria 1 and 2, we used a variety of key words (see electronic supplementary material, text S3) to search the Web of Science (www.webofscience.com) and Google Scholar (https://scholar.google.com), and checked the references in relevant publications. We also searched the Birds of the Western Palearctic [29], focusing on accounts of species where some information on timing of song or activity patterns was available. The data available from the literature were insufficient to conduct a formal review or meta-analysis. Instead, we describe and cite all relevant studies. However, most of the relevant evidence was anecdotal and often not the main focus of the publication, so it is likely that some publications were missed.

## 3. Results

At dawn, male blue tits sang more before their mate left the nest-box than afterwards (figure 1*c*; electronic supplementary material, figure S5, Poisson estimate ± SE: 1.34 ± 0.02, *p* < 0.001, *n* = 90 time periods from 45 visits by 27 females). During the day, males sang more when their female was inside the nest-box than during the same period before she entered or after she left the box (figure 1*d*; electronic supplementary material, figure S5; Poisson estimate ± SE: before—during: 0.37 ± 0.05, *p* < 0.001; during—after: −0.73 ± 0.06; *p* < 0.001, *n* = 165 time periods from 55 visits by 29 females). These results did not qualitatively change when the 2-min-periods just before or after female entry or exit were excluded (before—during: 0.27 ± 0.05, *p* < 0.001; during—after: −0.72 ± 0.06; *p* < 0.001, *n* = 127 time periods from 48 visits by 27 females). At dusk, males sang more after their social mate entered the nest-box (figure 1*e*; electronic supplementary material, figure S5; Poisson estimate ± SE: 5.38 ± 1.03, *p* < 0.001, *n* = 238 time periods from 119 visits by 65 females). However, this result should be interpreted cautiously, because males rarely sang at dusk (only *n* = 4 out of 119 produced song).

**Figure 2.**
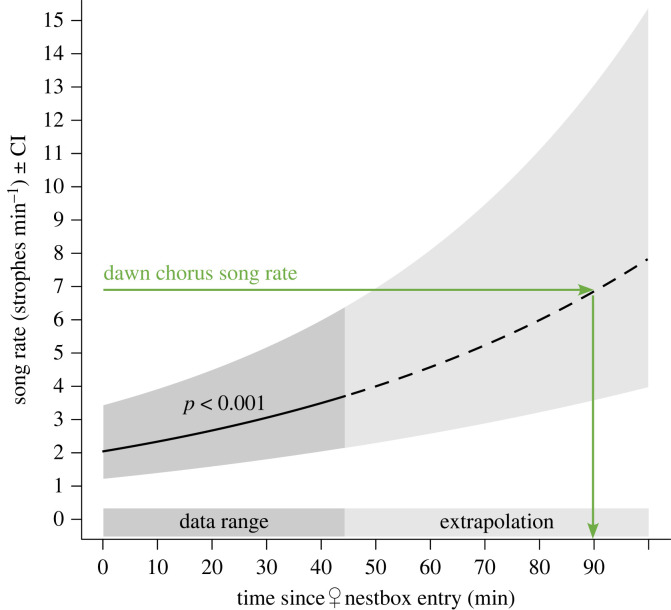
Male song rate (strophes min^–1^) increases with the duration of female absence. Shown are the model fit (line) and the confidence interval (CI, grey area) from a linear mixed-effect model based on data from male singing activity during female nest-box visits during the day. The horizontal green arrow indicates the average song rate observed during the dawn chorus. The vertical green arrow shows that, based on extrapolation of the model predictions, song rates would reach the level observed during the dawn chorus after a female absence of approximately 90 min.

Song rates during female absence were much higher at dawn than during the day or at dusk (figure 1*c–e*). Males increased their song rate with the time the female spent inside the nest-box (figure 2, estimate ± SE: 0.014 ± 0.002, *p* < 0.001, *n* = 1535 min based on 107 visits by 35 females).

## 4. Discussion

We examined the effect of female absence on male song rate during dawn, during the day, and at dusk (figure 1). In all periods, males sang more while their mate was inside the nest-box. The increase in song rate when the female was in the nest-box during the day indicates that male song output is higher whenever the social female is not with the male.

During the day, song rate increased the longer the female was inside the nest-box (figure 2). Within the first half hour of separation song rate increased by approximately 50%. If the increase in song rate can be extrapolated, song rates would reach the level of those observed during the dawn chorus after about 90 min of female absence (figure 2). This result can explain why males sing at a much higher rate at dawn after having been separated from their mate for an entire night than during the day or at dusk when their mate left only recently (figure 1). Our data on blue tits therefore support the ‘absent mate’ hypothesis.

### (a) How does the ‘absent mate’ hypothesis compare with other hypotheses about the dawn chorus?

The ‘absent mate’ hypothesis posits that males start singing or increase their song rate when they become separated from their social mate, without specifying why males do this. We assume that males benefit from keeping in contact with their mate (e.g. to look out for predators while foraging, for copulations and courtship feeding, to avoid take-over by another male, to avoid extra-pair copulations, or to strengthen the pair bond). Our hypothesis is different from—but does not contradict—the female manipulation hypothesis [3], because it does not hinge on the occurrence of a fertility window at dawn. Our hypothesis is also more general, because it applies independent of how males benefit from singing at dawn, whereby avoiding paternity loss (as under the fertility assurance hypothesis) may still be one of the key benefits.

A peak of singing at dawn may initially be adaptively neutral, but may acquire an adaptive function over time. For instance, the dawn chorus could convey information about the singing male's quality (‘honest signal of condition’ hypothesis [30,31]), or it could become important in territorial disputes (‘social dynamics’ hypothesis [32]; for a list of alternative hypotheses and additional factors that may shape the dawn chorus see electronic supplementary material, table S6). Thus, the ‘absent mate’ hypothesis predicts the presence (or absence) of a dawn chorus but does not exclude that the dawn chorus acquires additional functions (e.g. territory maintenance, attraction of extra-pair females) or that there are other benefits of singing at dawn (e.g. predation avoidance, low trade-off with foraging, optimal energetics and physical properties, ‘warm up’ before actual activity, stimulation of male or female; electronic supplementary material, table S6 in the electronic supplementary material). The ‘absent mate’ hypothesis is a more parsimonious and general explanation for the existence of a dawn (and dusk) chorus, because it does not require a special mechanism or evolutionary explanation. It is sufficient that many males are separated from their female simultaneously during a specific period of the day.

### (b) What does ‘female absence’ mean?

The ‘absent mate’ hypothesis suggests that the immediate trigger for higher male song output is the separation of the pair members, i.e. the perceived female's absence. We defined female absence as any time the female spent inside the nest-box, because during that time the male and female are not in immediate physical or visual contact. However, it remains unclear how the male perceives such a situation and how the song response is triggered. On the one hand, when the female is inside the nest-box, the male may be relieved of trade-offs between singing and social pair behaviour (e.g. mate-guarding). In this case, the male would increase his song rate because he is ‘free’ to shift his focus away from his mate.

On the other hand, female absence may trigger the male to re-establish contact with his mate. Males may then increase their song rate whenever their mate is out of visual contact to signal their presence or their location to their mate. Being in contact with the mate appears to be important in blue tits, because pair members often vocally interact with each other during the pre-laying and laying period [20,33]. The male might also perceive the female's absence as mate loss, particularly if he did not see her disappear in the nest-box and if she does not respond to his vocalizations.

The response of the partner to the absence of the mate does not need to be sex-specific. In fact, a previous study on blue tits showed that females also started to sing soon after their mate had been experimentally removed [33]. In general, immediate physical pair interactions such as courtship, copulation, courtship feeding or allopreening may be needed to ‘reset’ the perception of the intactness of the pair bond. Further study is necessary to investigate the proximate mechanism via which mate ‘absence’ is related to song output.

### (c) Can the ‘absent mate’ hypothesis also function in other species?

The ‘absent mate’ hypothesis is based on the idea that two simple criteria are sufficient to produce a dawn and dusk chorus. Criterion 1 implies a pair bond as well as a sex-difference in the start and end of daily activity, which could arise as a by-product of the influence of sexual hormones (such as testosterone) on daily rhythms [34,35]. Criterion 1 could therefore be valid across socially monogamous diurnal species. Criterion 2 implies that males respond to the absence of their social mate by increasing their song output or to the presence of their mate by reducing their song output. In many species, male singing is most pronounced during breeding [2] and specifically during the fertile period of the partner [3]. One likely function of song in addition to defending the territory and finding a partner is therefore that it serves to maintain the pair bond, and to avoid mate switching or loss of paternity [3]. Criterion 2 should thus be applicable to all species in which males use song to secure and maintain a social partnership. If song would solely be used in the context of male–male competition, criterion 2 may still be fulfilled, but only when the presence of the social female inhibits male song, for example because it trades off with other tasks (e.g. mate guarding [36]). In this case, song is suppressed during the presence of the social female and therefore the song rate will increase after the male and the female become separated. We note that our hypothesis can also apply to species that do not roost or nest in cavities, because nesting and roosting sites are generally hidden and the female on the nest is often cryptic to avoid predation (e.g. [37]). The ‘absent mate’ hypothesis can therefore theoretically be applied to the majority of species that produce a dawn chorus.

### (d) Is there empirical support for criteria 1 and 2 in other bird species?

To establish whether there is empirical support for the ‘absent mate’ hypothesis in other birds, we performed a literature search with a special focus on Passerines. Although the relevant information is mostly anecdotal, the existing data support the two fundamental criteria underlying the hypothesis in other species than the blue tit. Regarding criterion 1, there is circumstantial evidence that males of several passerine species are active earlier in the morning than females (e.g. great tit *Parus major* [21,38,39]; blue tit [40]; willow tit *Parus montanus* [41]; black-capped chickadee *Poecile atricapillus* [42]; American robin *Turdus migratorius* [43]; short-toed treecreeper *Certhia brachydactyla* [44]; wood warbler *Phylloscopus sibilatrix* [45]; Adelaide's warbler *Dendroica adelaidae* [46]; superb fairy wren *Malurus cyaneus* [47,48]) and that males sleep overall less than females [49]. Differences in the timing of activity between males and females could arise as a result of sex-specific diel changes in hormone levels, and could in particular lead to an earlier start of male activity in the morning [34,35]. We did not find any study on passerines showing either no difference in timing or females being earlier than males. The existing data thus support (and clearly do not contradict) the idea that an earlier start of activity in males (and possibly also longer activity in the evening) can be a general phenomenon among birds.

Regarding criterion 2, both descriptive and experimental studies on a variety of species have shown a relationship between male song output and female absence. Already in 1952, Hinde [21] described in great tits that ‘if the pair became separated during the pre-nesting period the male soon starts to sing’ (p. 69). He further noted that during the day, males showed similar vocal behaviour when the female was inside the nest-box as compared to dawn and dusk, especially ‘if the female stays unusually long in the box’ (p. 108). Other observations in great tits [22], common starlings *Sturnus vulgaris* [43,50], American robins [43], wood warblers [45], whited-throated sparrows *Zonotrichia albicollis* [51], zebra finches *Taeniopygia guttata* [52], Eastern bluebirds *Sialia sialis* [53], golden-winged warblers *Vermivora chrysoptera* [54] and screech owls *Otus asio* [55] indicate that male song rate increases in response to separation from the mate and decreases as soon as the pair is reunited. Furthermore, several experimental studies reported that males strongly increased their song rate during the day after their social mate was removed, and decreased their song rate again once the mate was returned (black-capped chickadee [56]; great tit [22,57]; common starling [50]; plain titmouse *Parus inornatus* [58]; zebra finch [59]*;* Adelaide's warbler [46]; yellow warbler *Dendroica petechia* [60,61]). We are not aware of any study showing that song rate does not change after temporary mate removal during the day (see also electronic supplementary material, text S4), suggesting that a link between male song output during the day and the absence of the mate may be a general phenomenon. Note that experiments carried out during the day typically removed the mate for a relatively short period. It therefore still needs to be established for a given species whether longer removal would lead to song rates that are as high as those observed during the dawn chorus. While mate removal affected song rates during the day, in several species it did not affect song rates at dawn (black-capped chickadee [42]; pied bush chat *Saxicola caprata* [62]; chipping sparrow *Spizella passerina* [63]*;* willow tit *Parus montanus* [64]; Adelaide's warbler [46]). In these experiments, males had been separated from their mate for the entire night either under natural conditions or when their mate had been experimentally removed. At dawn males may already sing at their maximum rates under natural conditions, in particular if song rate in these species increases with the time the female is absent (figure 2). Then, no further increase in song rates due to mate removal is expected.

### (e) Can the ‘absent mate’ hypothesis explain other aspects of singing?

A main strength of the ‘absent mate’ hypothesis is that it can help explain and predict various features of song, including daily and seasonal variation in song output and the presence or absence of a dawn and dusk chorus.

#### (i) Variation in the intensity of dawn and dusk song

The ‘absent mate’ hypothesis can explain why the dusk chorus of blue tits, and probably that of other species [3,20,43], is not as pronounced as the dawn chorus. At dawn, more males will be active simultaneously while their female is roosting than at dusk, because (1) in blue tits the time difference between male activity start and female emergence at dawn is on average three times as large (30 min) as the time difference between female roosting start and male activity end at dusk (10 min) [15] and (2) in blue tits and in other species the going-to-roost time at dusk is more variable among males than the start-of-activity time at dawn [2,15,21,43,44,65,66]. Additionally, if song rate increases with prolonged absence of the social mate, as we show for blue tits (figure 2), song rate should be higher at dawn than at dusk simply because of the difference in the duration of female absence.

#### (ii) Differences between dawn and day song

Several studies report differences between song produced during the day and dawn song, in particular in terms of song output [13,20]. The ‘absent mate’ hypothesis predicts such difference in song output, if male song rate increases with the time the female has been absent (as shown in figure 2). Long periods of separation of pair members are rare during the day, unless the female dies or deserts, whereas in many species these separations are the rule during the night. Variation in the duration of female absence may thus be sufficient to explain the strong difference between typical dawn and day song rates.

Several studies have also reported that males use different song types during the day than during the dawn chorus (e.g. [60,67–69]). While such differences are not predicted by the ‘absent mate’ hypothesis, they do not contradict it, because once a dawn chorus arises, the benefits of signalling and the information content of the signal may vary over time, potentially leading to the evolution of different song characteristics.

#### (iii) Daily variation in song output

The ‘absent mate’ hypothesis predicts that males should continue to sing at a high rate in the morning and throughout the day (similar to the rate observed during the dawn chorus) when their mate fails to appear from the night roost. The results of experiments in which females were kept in the nest-box or removed from the territory for a few hours after their normal emergence time qualitatively confirmed this prediction [42,53].

The hypothesis also predicts that during the day males should progressively increase their song rate after the disappearance of their mate. This prediction is clearly confirmed by previous studies that show a strong increase in song rate during the day after the female disappeared or was experimentally removed [22,50,56–59,70].

#### (iv) Seasonal variation in song output

Across species dawn song usually increases in the period leading up to breeding with a peak around the time of nest-building and fertilization [2], which is also observed in tropical and non-tropical species with year-round song [2,71–74(but see)]. Mechanistically, increased song rates may be a result of increased testosterone levels that induce changes in the brain's song nuclei [75–78]. Increased testosterone may simultaneously result in an earlier start of activity in males [34,35]. Females, on the other hand, likely exit their roost later during egg laying and incubation [15,79]. The ‘absent mate’ hypothesis predicts that the length of the dawn chorus should increase if earlier male and later female start of activity coincide, because this leads to a longer period of perceived ‘female absence’ in the morning. By linking song output to the duration of mate separation, the ‘absent mate’ hypothesis may thus also explain (1) why the intensity of daytime singing generally decreases after a pair is formed [45,57,80], (2) why it increases again during incubation [81–84] when the female is inside the nesting cavity [22], or when the male loses his mate [70,85] and (3) why, in contrast, dawn song rates generally remain high even after pair formation [2,38,86] (but see [87]).

#### (v) Dawn chorus in duetting species

In line with the ‘absent mate’ hypothesis, tropical species in which both pair members sing (duetting) usually do not have a pronounced dawn chorus [2]. In the rare cases where they do (e.g. in the buff-breasted wren *Thryothorus leucotis* [88] and the white-browed weaver *Plocepasser mahali* [89]), the dawn chorus is produced by the male [2]. The male-initiated dawn chorus may then be followed by a duet if the female joins the male after she emerges from the roost, which may in turn lead to a peak of duetting at dawn as observed in the rufous-naped wren [90].

#### (vi) Presence/absence of a dawn chorus

The ‘absent mate’ hypothesis predicts that any species in which individuals form pair bonds and in which both criteria are met should show a dawn chorus. The dawn chorus may thus not be limited to passerine birds, but may occur in other vocally active species (e.g. mourning dove *Zenaida macroura* [91]).

Based on the ‘absent mate’ hypothesis, we can make four testable predictions about the presence and absence of song ‘choruses’ in different species. (1) No pronounced dawn or dusk chorus should occur in species in which the male and the female of a pair spend the night together (e.g. long-tailed tit *Aegithalos caudatus* [16]; sand martin *Riparia riparia* [92]; goldcrest *Regulus regulus* [93]), at least if they enter and exit the roost together as well. (2) Species in which a male's vocal output is unrelated to the presence of the female, or is higher when the female is present, should not have a pronounced dawn chorus. This could for instance occur in colonial breeders, if males typically sing when in close contact with their mate or with competitors (e.g. ‘directed song’ of the zebra finch *Taeniopygia guttata* [59]). (3) Species exhibiting highly synchronous sex-specific behaviours that separate pair members at other times than at dawn (e.g. species in which females lay their egg later during the day), should show an intense bout of singing during that period of the day. For a chorus to arise, the duration of any behaviour that leads to separation of the pair members should be long enough to elicit high song rates and it should be synchronous between females. (4) Females should never produce a dawn chorus, even in species with pronounced female singing [94], unless they become active earlier in the morning than males. Three studies (on blue tits [33], great tits [21 p. 68] and European starlings [95]) describe that females sing if their social male is absent. The ‘absent mate’ hypothesis may therefore also be applicable to explain variation in female song output.

### (f) Conclusions

In their seminal review, Staicer *et al*. [2] proposed that ‘the focus on dawn chorus is rather narrow considering the sheer diversity of diel patterns and vocalizing among birds'. The ‘absent mate’ hypothesis addresses this issue in the sense that it explains not only the occurrence of the dawn (and dusk) chorus, but—more generally—diel and seasonal variation in song output. Indeed, the strength of this hypothesis is that it does not make specific assumptions about the importance of a particular time of day. Rather, it is based on the widely accepted view that song is used for mate attraction [2] in combination with an often reported difference in circadian timing between the sexes [21,38,39,41–45,47,48]. The ‘absent mate’ hypothesis leads to several clear and testable predictions about variation in song output. Although we show that the behaviour of blue tits conforms to the predictions of this hypothesis and find no observations from other species that refute it, rigorous tests of the ‘absent mate’ hypothesis across species are needed to evaluate its general applicability.

## Data Availability

All data and code have been deposited as a GitHub repository (https://github.com/lschlicht/DawnChorus), and are publicly available and archived at Zenodo [96]. Additional information is provided in the electronic supplementary material [97].
